# Is Cataract in Patients under 60 Years Associated with Oxidative Stress?

**DOI:** 10.3390/biomedicines11051286

**Published:** 2023-04-27

**Authors:** Hanna Lesiewska, Alina Woźniak, Paweł Reisner, Krzysztof Czosnyka, Joanna Stachura, Grażyna Malukiewicz

**Affiliations:** 1 Department of Ophthalmology, The Nicolaus Copernicus University, Ludwik Rydygier’s Collegium Medicum, 85-094 Bydgoszcz, Poland; 2 Department of Medical Biology and Biochemistry, The Nicolaus Copernicus University, Ludwik Rydygier’s Collegium Medicum, 85-094 Bydgoszcz, Poland

**Keywords:** presenile cataract, oxidative stress, antioxidant enzymes, lipid peroxidation

## Abstract

Oxidative stress is considered as a possible factor in the genesis of cataract. The study aimed to determine the systemic antioxidant status in cataract patients under 60 years. We studied 28 consecutive cataract patients, mean of 53 years (SD = 9.2), a range of 22–60 and 37 controls. In erythrocytes, activity of antioxidant enzymes was determined: superoxide dismutase (SOD), catalase (CAT), and glutathione peroxidase (GPx), in contrast with plasma concentrations of vitamin A and E. Conjugated dienes (CD) level and protein carbonyls (PC) concentration were also determined in plasma. Malondialdehyde (MDA) concentrations in erythrocytes and plasma were also measured. SOD and GPx activity and vitamin A and E concentrations were lower in cataract patients (*p* = 0.000511, 0.02, 0.022, and 0.000006, respectively). MDA plasma and erythrocytes concentrations were higher in cataract patients (*p* = 0.000001 and 0.0000001, respectively). PC concentration was higher in cataract patients than in controls (*p* = 0.00000013). There were statistically significant correlations between oxidative stress markers both in the cataract patients group as well as in the control group. Cataract incidence in patients under 60 years seems to be accompanied by enhanced lipid peroxidation and protein oxidation, as well as antioxidant defense depletion. Thus, supplementation with antioxidants could be beneficial in this group of patients.

## 1. Introduction

Cataract is the leading cause of blindness, affecting 46 million people worldwide [1,2]. Senile cataract is a progressive age-related disease characterized by gradual clouding of the lens and deterioration of vision. It is defined as cataract occurring in people >50 years of age, unrelated to known mechanical, chemical, or radiation trauma. It becomes progressively more severe and frequent in the elderly, responsible for 48% of world blindness [3,4]. Individuals with visually significant cataract tend to experience symptoms such as blurred vision, loss of contrast, halos, and difficulty with glare, which can considerably affect patients’ ability to perform day-to-day tasks [5]. It is widely accepted that oxidative stress is a significant factor in the genesis of experimental cataract, both in experimental animals and in cultured lens models [6,7,8,9]. The “oxidative stress theory” holds that a progressive and irreversible accumulation of oxidative damage caused by reactive oxygen species (ROS) impacts critical aspects of the aging process and contributes to impaired physiological function and increased incidence of diseases [10]. One theory postulated that in the aging eye, barriers develop that prevent glutathione and other protective antioxidants from reaching the nucleus in the lens, thus making it vulnerable to oxidation [11]. Some studies have revealed the increased lipid peroxidation products, which can be diffused from the retina to the lens through the vitreous body in human cataractous lenses [12]. Cataract becomes more common with age and multiple factors are suggested to be involved in the progression of lens opaqueness. Cataract is generally considered to be a usual age-related condition, but up till now, it is not clear why some individuals, otherwise healthy, have an increased risk of developing all types of cataracts at the age under 60 or even earlier.

The influence of oxidative stress on the pathogenesis of senile cataract has already been documented and is not questionable. Oxidative stress can damage tissues, leading to tissue structure and function changes, increased vascular permeability, microvascular abnormalities, and neovascularization. These changes can cause lens crystallin denaturation. There is a 30% decrease in serum TAC in cataract patients compared to healthy controls. Studies indicate that patients with cataracts have lower serum CAT and SOD levels than healthy controls. Examination of the serum CAT and SOD levels can be an important quantitative indicator for the clinical diagnosis of senile cataracts [13]. Oxidation-reduction processes may also be significant in the course of cataract in younger persons. Some research refers to this point, but those published so far that concern cataract in patients under 60 years of age indicate disorder of oxidant-antioxidant balance [14,15]. However, there is no definitive evidence that oxidative stress is a factor for cataract development in people of this age. Studies concerning redox balance may have practical implications in terms of the possibility of using antioxidants to reduce the risk of cataract formation or progression. Further research within this area is necessary as the published data concerning the advantages of antioxidant supplementation are still equivocal.

As it is becoming increasingly common for young individuals without chronic illnesses or risk factors to develop cataract, we decided to determine the systemic antioxidant status in this group of patients.

## 2. Materials and Methods

### 2.1. Subjects

We studied 28 consecutive patients (11 males and 17 females), aged under 60 years, who presented for cataract surgery, mean 53 years (SD = 9.2), range 22–60. The cataract was diagnosed by slit lamp examination after pupil dilation. Among the operated eyes, 8 nuclear, 14 cortical, and 6 posterior subcapsular cataracts were diagnosed. The visual acuity of at least one eye was equal or lower than 0.6 measured using the Snellen chart and presented in decimal values. In 20 patients, there was bilateral cataract in 8; the fellow eye was pseudophakic. The reference group consisted of 37 individuals without cataract matched for age and gender (Table 1). All subjects provided their informed consent for inclusion before participating in the study. The study was conducted in accordance with the Declaration of Helsinki, and the protocol was approved by the Ethics Committee of Collegium Medicum UMK (KB 866/18).

With the exception of cataract, the exclusion criteria included hypertension, diabetes, rheumatoid arthritis, lipid profile disorders, autoimmune diseases, neurologic disorders, glaucoma, myopia over 5 diopters, intraocular surgery in history, age-related macular degeneration, optic neuritis, chronic inflammatory ocular conditions, previous ocular trauma, and Sjögren syndrome [13]. Excluded from the study also were subjects smoking cigarettes as well as those burdened with medical conditions during which occurrence of oxidative stress had been proven. No patients were declaring excessive alcohol intake of more than 7 drinks for women and more than 14 drinks for men per week. Performing physical activity was not an exclusion criterion for this study. As some authors have stated that there is a modest relationship between body mass index (BMI) and the plasma lipid peroxidation level, we calculated the BMI of our patients [16]. The mean BMI was 26.3 in the studied group, and 25.7 in the control group (*p* = 0.32). Blood pressure was also not significantly different between the two groups (Table 1).

### 2.2. Measurement of Oxidative Stress Parameters

The activities of antioxidant enzymes such as SOD, CAT, and GPx were measured in erythrocytes. The concentrations of vitamin E (α-tocopherol), vitamin A (retinol), as well as CD and PC were measured in plasma. The level of MDA was quantified in erythrocytes and plasma samples.

A whole blood sample with the addition of K_2_EDTA was centrifuged at 4075× *g*, 4 °C for 10 min. The upper layer of plasma was aspirated and stored at −20 °C for further analyses. Leukocytes were discarded and red blood cells were washed twice with a phosphate-buffered saline (PBS) without calcium and magnesium ions (Biomed, Lublin, Poland). Erythrocyte mass was suspended in PBS (1:1 by volume) and used for further analyses.

The hemoglobin concentration was measured by the standard colorimetric method using Drabkin’s reagent and expressed as g⁄dL.

#### 2.2.1. Determination of Superoxide Dismutase Activity

SOD activity was assayed by the method of Misra and Fridovich [17]. In this method, SOD activity is measured as an ability to inhibit the autooxidation of adrenaline in an alkaline solution. Briefly, 100 µL of the suspension of erythrocytes in PBS (1:1) was diluted 10 times with deionized water, and then 500 µL of ethanol and 250 µL of chloroform were added. The mixture was vortexed for 3 min and centrifuged at 13,680× *g*, 4 °C for 10 min and the resulting supernatant was collected. An enzymatic reaction mixture consisted of 3.3 mL of 40 mM carbonate buffer (pH 10.2) containing 0.136 mM EDTA, 100 µL of supernatant, and the reaction was initiated by adding 200 µL of 9 mM adrenaline in 10 mM HCl. The reference reaction mixture contained 100 µL of deionized water instead of the supernatant. The reactions were performed in spectrophotometric cuvettes at 36.7 °C and the adrenochrome formation was monitored for 3 min at 480 nm. The rate of adrenochrome formation was calculated based on the slope of the linear part of adrenochrome formation curve within the same time interval in the case of the reference and sample reaction. SOD activity unit was defined as the amount of activity which inhibits the adrenaline autooxidation rate down to 50% of that observed in the reference reaction. Specific SOD activity was expressed as a number of U per g of Hb.

#### 2.2.2. Determination of CAT Activity

The CAT activity was measured according to the method of Beers and Sizer [18]. Briefly, 100 µL of erythrocyte mass was diluted 1000 times with deionized water. The enzymatic reaction was performed in a quartz spectrophotometric cuvette by adding 200 µL of the hemolysate to 2.5 mL of 50 mM phosphate buffer (pH 7.0) containing 50 mM H_2_O_2_. A decrease in H_2_O_2_ concentration was monitored spectrophotometrically for 30 s at 240 nm, 36.7 °C. CAT activity unit (U) was defined as the amount of activity decomposing 1 μmol of H_2_O_2_ per min. Specific activity was expressed as a number of U per g of hemoglobin (Hb).

#### 2.2.3. Determination of GPx Activity

GPx activity was measured according to the method of Paglia and Valentine [19]. In this method, the sample GPx decomposes the substrate H_2_O_2_ using the reduced form of glutathione (GSH). The oxidized glutathione (GSSG) formed in the reaction mixture is then converted back to GSH form by the excess of externally added glutathione reductase (GR) along with its coenzyme, reduced nicotinamide adenine dinucleotide phosphate (NADPH). An oxidation of NADPH to NADP^+^ is monitored spectrophotometrically to determine its initial rate in the reaction mixture. Stoichiometrically, the decomposition of one mole of NADPH is equivalent to the reduction of one mole of H_2_O_2_. Since a noticeable oxidation of NADPH occurs in this system without investigated hemolysate, the rate of this GPx-independent reaction must be measured in a reference reaction mixture and later subtracted from the rate observed in the sample reaction.

Briefly, 100 µL of suspension of erythrocytes in PBS (1:3) was added to 400 µL of deionized water, vortexed, capped, and placed in −20 °C for 4 min, while avoiding freezing. The sample tube was centrifuged at 4075× *g*, 4 °C for 10 min. A total of 100 µL of resulting supernatant was mixed with 100 µL of Drabkin’s reagent and assayed for GPx activity. The enzymatic reaction mixture (total volume of 3 mL) contained 73.3 μM H_2_O_2_, 44 mM phosphate buffer (pH 7.0), 440 µM EDTA-Na_2_ × 2 H_2_O, 9.33 µM NADPH-Na_4_, 3.75 mM NaN_3_, 5 mM GSH, 1 U of GR, 50 µL of the assayed hemolysate. The reaction was initiated by adding H_2_O_2_ solution, and the rate of NADPH oxidation was determined by measuring the absorbance changes at 340 nm, at 20 °C, between the 2nd and 4th min after initiation. In the reference reaction, the hemolysate was substituted with 50 μL of deionized water. GPx activity unit (U) was defined as the amount of activity converting 1 μmole of NADPH to NADP^+^ per min. Specific activity was expressed as number of U per g of Hb.

#### 2.2.4. Determination of MDA Concentration in Plasma and Red Blood Cells

Briefly, 100 µL of 0.01% butylated hydroxytoluene (BHT) in acetone and 500 µL of 5% trichloroacetic acid (TCA) were added to 500 µL of hemolysate (0.25 mL of suspension of red blood cells: 0.25 mL H_2_O), and incubated at RT for 10 min. Then, the sample was centrifuged at 4000× *g*, for 10 min. 500 µL of obtained supernatant or 500 µL of plasma was added to 4.5 mL of 0.375% thiobarbituric acid (TBA) in 15% TCA and incubated at 100 °C in the water bath for 20 min. The cooled samples were centrifuged at 12,000× *g*, 4 °C for 10 min. A 50 µL aliquot of obtained supernatants was chromatographed on the Symmetry C18 column (150 × 3.9 mm, 5 µm grain, Waters, Milan, Italy) by high-performance liquid chromatography (HPLC) with fluorescence detector (ProStar 210 Pump, 363 Fluorescence Detector, Varian, Palo Alto, CA, USA). The column was eluted at 1 mL/min with 50 mM ammonium formate (pH 6.5) and methanol (60:40, *v*/*v*). The excitation and emission wavelengths were 532 and 553 nm, respectively [20].

#### 2.2.5. Determination of Vitamin A and E Concentrations in Plasma

The plasma concentrations of vitamin A (retinol) and E (α-tocopherol) were quantified using the HPLC method with UV detection (ProStar 325 UV-Vis detector, Varian, USA). Briefly, 800 µL of acetonitrile was added to 200 µL of plasma sample, shaken for 2 min, and centrifuged at 10,000× *g*, 4 °C for 10 min. Then, the supernatant was extracted with 4 mL of hexane. The hexane fraction with vitamins was evaporated to dryness under a nitrogen atmosphere at 40 °C and dissolved in 100 µL of the mobile phase consisting of acetonitrile: methanol (95:5, *v*/*v*). A 10 μL aliquot of the sample was chromatographed at a flow rate of 2 mL/min on the Kinetex C18 column (75 mm × 4.6 mm, 2.6 μm grain, Phenomenex, Torrance, CA, USA) coupled to the HPLC system. Vitamins A and E were quantified by integration of chromatograms acquired at 325 and 295 nm, respectively. The concentrations of vitamins are expressed in micrograms per liter of plasma (µg/L). The quantitative determinations were performed using calibration curves for retinol and α-tocopherol with their acetate derivatives as internal standards [21,22].

The level of CD was expressed in absorbance units per milliliter of blood plasma (Abs./mL). PC concentration was indicated by derivatizing samples with dinitrophenylhydrazine (DNPH) and measuring the bound anti-DNPH antibodies using microplate reader ELISA and readymade reagent sets, and was expressed in U/mL [23].

### 2.3. Statistical Analysis

The statistical analyses were performed with Statistica 13.1 software (TIBCO Software, Palo Alto, CA, USA). The Student’s *t*-test was used for independent samples assuming equal or unequal variances (Levene’s test). Only GPx, vitamin E, and PC did not meet the basic criterion of the test (normality in Kolmogorov–Smirnov test); therefore, results of that parameter were evaluated with the Mann–Whitney test. Linear relationships between parameters were additionally analyzed in the study, Pearson’s r coefficient was calculated, and a multiple linear regression model with a stepwise method was performed. The *p*-value of <0.05 was considered statistically significant. The results are shown as arithmetic means ± standard deviations.

## 3. Results

**Figure 1:**
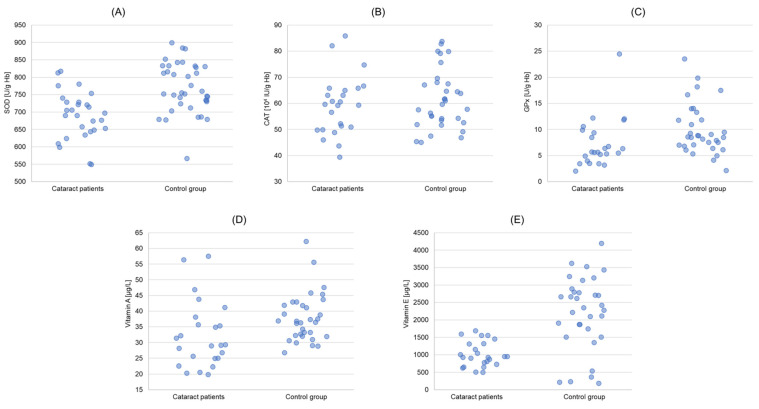
Charts presenting the results obtained for the group with cataract and the control group. (**A**): superoxide dismutase (SOD) activities in erythrocytes; (**B**): catalase (CAT) activities in erythrocytes; (**C**): glutathione peroxidase (GPx) activities in erythrocytes; (**D**): vitamin A concentrations in blood plasma; (**E**): vitamin E concentrations in blood plasma.

**Figure 2:**
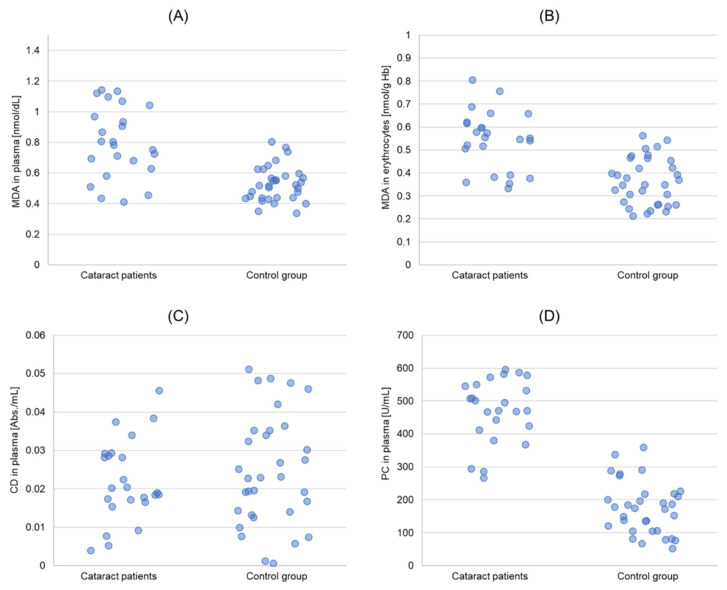
Charts presenting the results obtained for the group with cataract and the control group. (**A**): malondialdehyde (MDA) concentrations in erythrocytes; (**B**): MDA concentrations in plasma; (**C**): conjugated dienes (CD) levels in erythrocytes; (**D**): protein carbonyls (PC) levels in blood plasma.

The antioxidant enzymes activity in the erythrocytes, concentration of antioxidant vitamins in plasma, as well as the concentration of MDA in plasma and erythrocytes, level of CD, and concentration of PC in plasma are presented in Table 2 and Table 3, as well as Figure 1 and Figure 2.

The statistical significance was revealed for SOD and GPx activities, vitamin A and vitamin E concentrations, as well as MDA plasma and erythrocytes concentrations and PC concentration between studied and control groups. The SOD activity in erythrocytes of cataract patients was 9.6% lower (*p* = 0.000511), and that of GPx, 23.5% lower (*p* = 0.02) than in controls. The CAT antioxidant activity was not statistically different between the studied groups. Vitamin A concentration was 15% lower (*p* = 0.022), and that of vitamin E, 59.8% lower (*p* = 0.000006) in cataract patients than in controls (Table 2). The MDA concentration, however, was higher in cataract patients than in the control group: 50.1% in plasma (*p* = 0.000001) and approximately 49.2% in erythrocytes (*p* = 0.0000001), respectively (Table 2). PC concentration in plasma was about 3.3 times higher in cataract patients than in the control group (*p* = 0.00000013). CD level in plasma was comparable in both groups (Table 3).

**Figure 3:**
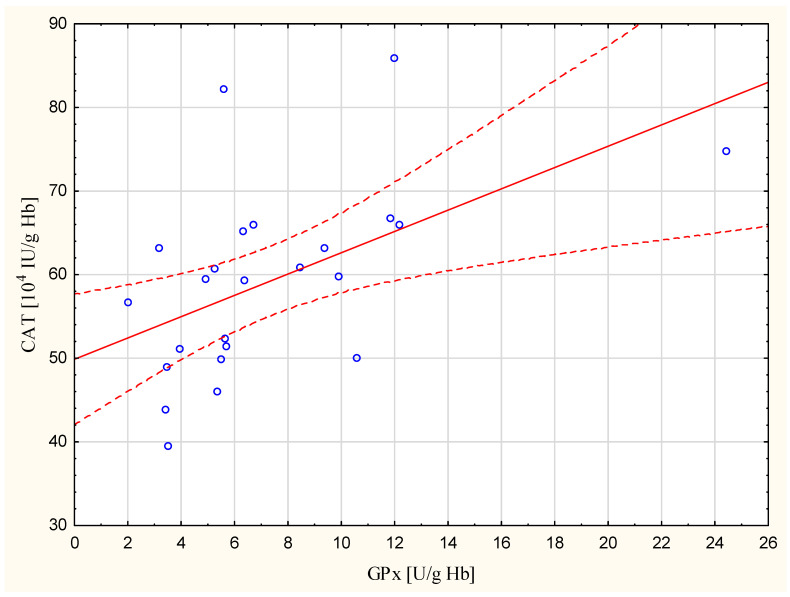
Linear regression of the catalase (CAT) activity versus glutathione peroxidase activity (GPx) in the erythrocytes of patients with presenile cataract (r = 0.530, *p* = 0.008).

Neither group had a statistically significant correlation between age and antioxidant enzymes activity. There was no statistically significant correlation between age and MDA concentration, either. It was demonstrated, however, that there were statistically significant correlations between the designated oxidative stress markers both in the cataract patients group as well as in the control group (Table 4 and Figure 3).

The stepwise multiple linear regression analysis confirmed what previous statistical tests had shown. Namely, belonging to the group of patients with cataracts was associated with lower SOD and GPx activities as well as vitamin A and E concentrations compared to the control group. An opposite trend was observed for plasma and erythrocyte MDA concentrations and plasma PC concentration. Moreover, the study participants significantly differed in terms of vitamin A concentration and SOD activity depending on gender, with GPx activity depending on the value of diastolic blood pressure. The final multiple linear regression model is presented in Table 5.

## 4. Discussion

It is commonly known that cataract is a prevalent result of aging. Biologic age refers to changes in the body that commonly occur as people age. Because these changes affect some individuals earlier than others, some people are biologically old at 65, while others are not until another decade or more. However, the most noticeable differences in apparent age among people of comparable chronologic age are caused by lifestyle, habit, and subtle effects of disease. According to some studies, aging is associated with elevated oxidative stress markers and disturbed antioxidant defense enzymes activity [10,24]. There is increasing evidence that oxidative stress has been implicated in the development of experimental cataract [6,7,8,9].

In our study, MDA plasma and erythrocytes concentrations and PC plasma concentration were higher in cataract patients than in controls (Table 3), which indicates that the occurrence of cataract before 60 is accompanied by the enhancement of the process of lipid peroxidation and protein oxidation. It is not possible to unequivocally conclude that the observed higher intensity of lipid peroxidation and protein oxidation was the cause of cataract occurrence. However, it is difficult to exclude such a possibility, even more so as it is also indicated by multiple linear regression (Table 5). Numerous studies confirm that crystallins undergo extensive oxidative modifications [25,26]. PC are commonly used for estimating the degree of oxidative damage of proteins. Higher concentration of PC in the plasma of patients with age-related cataract is also confirmed by the studies of Chang et al. [26]. A number of studies indicated a higher concentration of MDA and CD in patients with age-related cataract. CD and MDA are the only markers of the lipid peroxidation process. CD are generated at the beginning of the lipid peroxidation process, as a result of hydrogen-atom detachment from the rest of polyunsaturated fatty acids and rearrangement of double bonds [27]. In the present paper, the level of CD in the plasma of cataract patients was, however, approximate to the level measured in healthy subjects. MDA is a secondary product of the lipid peroxidation process, developing as a result of decomposition of arachidonic acid and larger PUFAs [28]. It reacts with numerous biomolecules, among others, with DNA and proteins, which leads to the production of adducts [28,29]. MDA adducts may participate in many secondary reactions leading to, among others, intramolecular or intermolecular protein/DNA crosslinking, which changes the biochemical properties of biomolecules [28,29]. Significant enhancement of the lipid peroxidation process may directly damage cell membranes, thus leading to distortion of cell functioning and cell death [30,31]. In the case of conventional age-related cataracts, the lipid peroxidation process is regarded as one of the processes of cataractogenesis [32]. In the aging eye, enhancement of lipid peroxidation may occur as a result of enhanced promotion of oxygen free radicals in the eye fluids and tissues and the weakened antioxidative defense of the crystalline lens [33]. It is possible that the lipid peroxidation process is one of the mechanisms responsible for cataract occurrence not only in the elderly, but also in persons below the age of 60 who are healthy and not burdened with additional medical conditions. It is hard to indicate a cause for distortion of the oxidant-antioxidant balance in these persons. These are not medical conditions during which enhanced development of ROS was proven, because such persons were excluded from the study. However, it should be remembered that an enhanced ROS generation not only accompanies the medical conditions, but it may also, for instance, be associated with physical activity. It was proven that lack of physical activity leads to oxidative stress [34], but intensive physical effort may also distort oxido-reductive processes [27]. Screening for presenile nuclear cataract indicates, among others, lack of exercise or high amount of physical exercise as risk factors for the occurrence of this medical condition [35].

The activity of SOD and GPx in erythrocytes in our study was lower in cataract patients when compared to the control group (Table 2). Vitamin A and E concentrations in plasma were also lower in cataract patients. The tendency to lower levels of these markers in patients with cataracts is also suggested by the multiple linear regression model obtained in the study (Table 5). The activity of CAT, however, did not differ in the studied groups in a way that would be statistically significant. Lower activity of SOD, GPx, and lower concentrations of vitamin A and E in cataract patients than in the control group, with simultaneously higher concentration of MDA in plasma and erythrocytes and PC in plasma, indicates the distortion of the oxidant-antioxidant balance, as a result of lowering the enzymatic activity of the antioxidant barrier and enhanced use of antioxidant vitamins. SOD, GPx, and CAT are the three main enzymatic systems of defense against free radicals and peroxides. SOD is an enzyme participating in the dismutation of superoxide anion radical (O_2_^•−^) into oxygen and hydrogen peroxide, whereas GPx and CAT are responsible for the disposal of hydrogen peroxide [36]. Simultaneous involvement of both enzymes in the disposal of hydrogen peroxide produced in excess has been revealed in our cataract patients with indicated positive and statistically significant correlation between the activity of GPx and CAT in erythrocytes (r = 0.530, *p* = 0.008) (Table 3, Figure 1). A similar correlation was not demonstrated in the control group. Available data show that no general tendency of evolution of these systems in aging emerges, even if some studies in humans demonstrate the existence of a concomitant decrease in most of the antioxidant enzymes in the blood of the elderly [14]. Our results showed no correlation between age and SOD, CAT, and GPx activity in both studied groups. There was no correlation between age and MDA levels both in cataract patients and the control group, either. At the same time, by using multivariable linear regression with the stepwise method, we proved that gender of the human subjects might have a significant impact on plasma vitamin A concentration and erythrocyte SOD activity, in contrast with diastolic blood pressure on erythrocyte GPx activity (Table 5). The study of Gartaganis et al. that did not prove the correlation between age and lipid peroxidation markers confirms our results [37]. Vitamins which are non-enzymatic antioxidants have an advantageous impact on maintaining the structural integrity and function of the lens [38]. Based on meta-analysis, it has been proven that a high level of vitamin E in blood serum might be significantly associated with reduced age-related cataract risk [39]. Earlier studies of different authors indicate, in turn, that supplementation of α tocopherol and β carotene does not have a protective impact on the incidence of cataract extraction in male smokers (both in the case of presenile and senile cataract) [40]. However, some other studies indicate that low serum levels of tocopherol and β-carotene are significantly associated with an increased risk for senile cataracts [13].

The majority of current research focuses on the molecular processes of redox biology in the development of senile cataract. The majority of such studies includes people over the age of 60. In the POLA study concerning the population of 60–95 years old, the association between cortical cataract and high red blood cell SOD activity was found [41]. AREDS study refers to a group between 60 and 80 [42]. The average age of individuals included in our study was much lower, with a mean age of 53 years. There are few studies available in which the authors analyzed oxidative stress markers in presenile cataract patients, otherwise healthy subjects not burdened with additional medical conditions, and in addition, the results presented are equivocal. In the study of Li et al. [12], there was no significant difference in plasma antioxidants, total antioxidant performance, and lipid peroxidation determined by MDA between the groups with and without early cataract. Nevertheless, the authors concluded that subjects with early cataract are under systemic oxidative stress, which can be identified by a sensitive biomarker of lipid peroxidation such as isomers of hydroxy octadecadienoic acid. In turn, other studies demonstrate lower plasma antioxidant capacity, but higher SOD and CAT activity in erythrocytes of presenile and senile cataract patients in comparison with healthy persons [14]. Analyzing oxidative stress markers in patients with presenile and senile cataract, Virgolici et al. did not manage to indicate differences in CAT and SOD activity, nor the differences in total antioxidant capacity and plasma residual antioxidant capacity between the abovementioned groups of patients [14]. In contrast, TBARS concentration in plasma was significantly statistically higher in patients with presenile cataract in comparison with senile cataract patients. The concentration of thiobarbituric acid reactive substances (TBARS) in plasma was significantly higher than in healthy subjects only in the case of patients with presenile cataract. Higher MDA concentration in cataract patients aged 52 ± 8 and lower glutathione level compared with healthy controls are confirmed by the studies of Kharb et al. [15]. Other authors also confirmed a higher level of MDA in the plasma of cataract patients compared to results obtained in subjects without cataract [43]. When comparing MDA concentrations in the plasma of cataract patients separated into two age groups, 40–60 and 61–80 years old, these investigators found no statistically significant changes. No differences were indicated either in PC concentration in plasma of patients between the above groups [43].

Lower activity of SOD, GPx, and CAT in erythrocytes was in turn demonstrated by Chandrasena et al. in senile cataract patients compared to the non-cataract patients [44]. The results of the prospective study of Delcourt et al. suggest that antioxidant enzymes SOD and GPx might be implicated in the etiology of cataract in people at the age of 60 or older [41]. The authors found that the incidence of cataract was increased in subjects with high red blood cell SOD and high plasma GPx activities. In another study, in the persons aged 45–75 years with cataract, similar to the present authors’ studies, the higher concentration of MDA in blood serum was observed as well as lower SOD activity in blood serum and GPx indicated in the whole blood, compared to controls [45]. It seems that the observed differences between the authors’ results analyzed in this paper and those presented in the available literature appear to be related to the choice of the studied group, such as the age range (wide or narrow) in which the participants in the study were included, or the stage of cataract advancement.

A certain limitation for the studies of the present paper was an indication of only a part of significant oxidative stress markers. The main reasons for not succeeding in indicating a greater number of markers, which would positively influence the interpretation of the results obtained, allowing for a more precise characterization of the oxidant-antioxidant balance in the course of cataract in patients under 60 years, were financial considerations and a low amount of venous blood drawn for the current study. Taking into account the limitations mentioned above, the present study, however interesting it may be, should be treated as a pilot study. Nevertheless, this study provided the validation of knowledge gained from a limited number of research studies on free radical processes in young individuals with cataract, as well as the assessment of the effectiveness of the research methodologies used herein.

It is believed that with the use of antioxidant biomarkers, patients with a low antioxidant capacity can be identified and antioxidant supplementation might be implicated and used for disease prevention or treatment [13]. Natural antioxidant therapy has been reported for various ophthalmic diseases, such as dry eye, cataracts, glaucoma, and AMD. A large-scale clinical trial provided by the National Eye Institute (USA) on vitamin C, vitamin E, and beta-carotene supplementation among 3640 AMD patients and 4629 cataract patients found that antioxidant treatment significantly affected cataract and effectively slowed AMD progression [46]. There is a hope that some nutritional and metabolic antioxidants may one day be effective in delaying or even preventing cataract formation in humans [7].

## 5. Conclusions

The preliminary results obtained in this study suggest that in cataract patients under 60 years, enhancement of lipid peroxidation and protein oxidation occurs. At the same time, lower SOD and GPx activity in erythrocytes and lower vitamin A and E concentrations in cataract patients than in the control group may indicate a distortion of balance between ROS generation and their disposal. The reason for this disturbance seems to be the weakening of the enzymatic barrier and enhanced vitamin use. Thus, supplementation with antioxidants could be beneficial in this group of patients. Our findings may shed light on the development of cataract in young individuals; however, due to the small number of patients included in this study, more research concerning this issue is required.

## Figures and Tables

**Table 1:** Clinical characteristics of cataract and control groups.

Demographics	Cataract Patients	Control Group
N	28	37
Age (years)	53 ± 9.2	53 ± 9.3
Sex (F/M)	17/11	22/15
BMI	26.3 ± 3.1	25.7 ± 3.8
Systolic blood pressure (mm Hg)	129 ± 14	131 ± 13
Diastolic blood pressure (mm Hg)	78 ± 6	78 ± 8

Data are means ± SD.

**Table 2:** Antioxidant barrier parameters in cataract and control groups.

Enzyme	Cataract Patients	Control Group	*p*
SOD (U/g Hb)	689.88 ± 74.47	763.52 ± 77.62	0.000511
CAT (10^4^ IU/gHb)	59.22 ± 11.35	60.91 ± 10.90	0.56
GPx (U/g Hb)	7.34 ± 4.72	9.60 ± 4.76	0.02
Vitamin A (µg/L)	32.40 ± 10.56	38.26 ± 7.75	0.022
Vitamin E (µg/L)	919.04 ± 330.85	2288.23 ± 1044.12	0.000006

SOD: superoxide dismutase; CAT: catalase; GPx: glutathione peroxidase.

**Table 3:** The level of lipid and protein oxidation products in cataract and control groups.

	Cataract Patients	Control Group	*p*
MDA in plasma (nmol/dL)	0.803 ± 0.231	0.535 ± 0.113	0.000001
MDA in erythrocytes (nmol/gHb)	0.546 ± 0.128	0.366 ± 0.099	0.0000001
CD in plasma (Abs./mL)	0.022 ± 0.010	0.025 ± 0.010	0.2725
PC in plasma (U/mL)	475.30 ± 136.41	142.17 ± 47.63	0.00000013

MDA: malondialdehyde; CD: conjugated dienes; PC: protein carbonyls.

**Table 4:** Statistically significant correlation coefficients between the parameters measured in the cataract patients and in control group.

Parameters	r	*p*
Cataract patients		
MDA in plasma/MDA in erythrocytes	0.438	0.032
GPx/CAT	0.530	0.008
SOD/GPx	0.429	0.037
CAT/PC	−0.663	0.004
CD/PC	0.629	0.007
Control group		
SOD/GPx	0.408	0.012

**Table 5:** Multiple linear regression. Blank spaces result from stepwise method.

	SOD (U/g Hb)	CAT (10^4^ IU/g Hb)	GPx (U/g Hb)	Vitamin A (µg/L)	Vitamin E (µg/L)	MDA in Plasma (nmol/dL)	MDA in Erythrocytes (nmol/g Hb)	CD in Plasma (Abs./mL)	PC in Plasma (U/mL)
	Coef./*p*-Value	Coef./*p*-Value	Coef./*p*-Value	Coef./*p*-Value	Coef./*p*-Value	Coef./*p*-Value	Coef./*p*-Value	Coef./*p*-Value	Coef./*p*-Value
Study group (1—cataract patients, 0—control group)	−0.82/<0.001	−0.17/0.419	−0.52/0.031	−0.47/0.029	−0.98/<0.001	1.33/<0.001	1.20/<0.001	−0.20/0.447	1.05/<0.001
Gender (1—Male, 0—Female)	−0.74/0.001			0.48/0.033			−0.34/0.111		
Age (years)	0.19/0.067		0.22/0.057		0.18/0.12				−0.22/0.071
BMI (kg/m^2^)		0.18/0.192		0.09/0.46		−0.10/0.307			
Systolic blood presure (mm Hg)		−0.21/0.146			−0.07/0.535		−0.10/0.313	−0.05/0.665	
Diastolic blood presure (mm Hg)		0.16/0.195	−0.24/0.044	−0.19/0.106		0.18/0.055		0.22/0.082	0.18/0.167

## Data Availability

Data supporting reported results can be found at the Department of Ophthalmology, University Hospital no. 1, Bydgoszcz, Poland.
